# Identification of Genes Related to White and Black Plumage Formation by RNA-Seq from White and Black Feather Bulbs in Ducks

**DOI:** 10.1371/journal.pone.0036592

**Published:** 2012-05-15

**Authors:** Shijun Li, Cui Wang, Wenhua Yu, Shuhong Zhao, Yanzhang Gong

**Affiliations:** Key Lab of Agriculture Animal Genetics, Breeding, and Reproduction of Ministry of Education, Huazhong Agricultural University, Wuhan, People's Republic of China; Arizona State University, United States of America

## Abstract

To elucidate the genes involved in the formation of white and black plumage in ducks, RNA from white and black feather bulbs of an F_2_ population were analyzed using RNA-Seq. A total of 2,642 expressed sequence tags showed significant differential expression between white and black feather bulbs. Among these tags, 186 matched 133 annotated genes that grouped into 94 pathways. A number of genes controlling melanogenesis showed differential expression between the two types of feather bulbs. This differential expression was confirmed by qPCR analysis and demonstrated that *Tyr* (Tyrosinase) and *Tyrp1* (Tyrosinase-related protein-1) were expressed not in W-W (white feather bulb from white dorsal plumage) and W-WB (white feather bulb from white-black dorsal plumage) but in B-B (black feather bulb from black dorsal plumage) and B-WB (black feather bulb from white-black dorsal plumage) feather bulbs. *Tyrp2* (Tyrosinase-related protein-2) gene did not show expression in the four types of feather bulbs but expressed in retina. *C-kit* (The tyrosine kinase receptor) expressed in all of the samples but the relative mRNA expression in B-B or B-WB was approximately 10 fold higher than that in W-W or W-WB. Additionally, only one of the two *Mitf* isoforms was associated with plumage color determination. Downregulation of *c-Kit* and *Mitf* in feather bulbs may be the cause of white plumage in the duck.

## Introduction

Identification of genes controlling plumage color and their associated inheritance patterns are important topics in poultry science research. Plumage color control is essential for the uniform appearance of birds in the poultry industry. White plumage is the most favorable color for producers of meat-type commercial birds not only because ducks with unpigmented feathers are easy to clean (comparing with unpigmented feather body, pigmented feather bulbs or follicles left in skin showing black dots and make carcass look dirty ), but also genes involved in melanogenesis may have pleiotropic effects on other phenotypes [1]. It has been reported that multiple genes exist at different loci controlling plumage color in ducks [2]. These loci include white neck MR, extended black E, blue dilution G, dominant white belly S, head cheek decorated R, white skin and mouth Y, and recessive white c. Compared to studies of plumage color in chicken and quail, few gene or pathway identification studies have been conducted in ducks. A 6-bp deletion that inactivated Tyrosinase (*Tyr*) in a line of albino chickens was reported [3]. Chang et al. [4] found that the causal mutation for the recessive white allele in chickens is the insertion of a complete avian retroviral sequence in intron 4 of the tyrosinase gene. The *Mitf* (Microphthalmia-associated transcription factor) gene encodes a transcription factor of *Tyr* family genes with important roles in pigmentation. MITF seems to be primarily associated with loss of pigmentation and patterning, i.e., white spotting in both dogs and cattle [5]–[7] as opposed to hyperpigmentation, which in the Silky was recently shown that the higher expression of *Mitf* is a downstream effect of increased EDN3 expression [8]. Higher expression of *Mitf*, which is associated with hyperpigmentation, was observed in Silky Fowl [9]. A stop codon caused by a 2-bp deletion in exon 11 of *Mitf* was found to be responsible for the “silver” plumage color in Japanese quail [10]. *Mitf* expression can be regulated by *Scf*-*Kit* signaling and can itself activate the transcription of the *Tyr* genes [11], [12]. *c-Kit* is required during the feather growth cycle for melanocyte activation in humans [13]. Mutations in c-*Kit* can cause coat color change in mammals [14]–[16]. Allele-specific genetic interactions between *Mitf* and *c-Kit* were also reported to affect melanocyte development in humans [17]. The expression pattern of *c-Kit* was investigated during embryonic development in chicken and quail [18], [19]. Mutations in other genes were also found to be associated with plumage color in these systems. Gunnarsson et al. [20] reported an 8.3-kb deletion upstream of *Sox10* that caused dark-brown plumage in chickens. Other genes, including *Mc1r*, *Asip*, and *Pmel17*, also contribute to plumage color [21]–[23]. Few recent studies have focused on the genetic mechanisms involved in duck plumage color formation. High-throughput genomic approaches are promising ways to identify genes and pathways involved in plumage color formation. Due to the unavailability of an assembled reference sequences, high-throughput expression tools have not been widely used in ducks, although one study used chicken microarrays for genome-wide expression analysis to identify genes related to sperm storage [24].

The white *Liancheng* is an egg-type duck and white *Kaiya* is a meat-type duck in South China. In our previous study, 80% of individuals in an F_1_ population from a *Kaiya* × *Liancheng* cross had a phenotype of grey plumage on their heads, wings, backs or tails, with a white belt running from neck to chest. The F_2_ population was segregated, individuals with white, black, and black-white plumage were found. We reported a new autosomal locus (designated T) that may control plumage color in ducks [25]. However, the identity and number of genes involved in plumage color control in these ducks is not clear.

This study is the first genome-wide expression analysis to use RNA-Seq to find differentially expressed genes related to black and white plumage color in ducks. A large number of genes was found to be differentially expressed between white and black feather bulbs. Our analysis found that important genes and pathways associated with pigment formation are differentially regulated between black and white feather bulbs. We further characterized the expression of a few key genes related to pigmentation.

## Results

### Overview of RNA-Seq Data

To maximize the coverage of duck feather bulb mRNA by RNA sequencing, libraries were constructed by pooling RNA isolated from 6 white feather bulbs (3 from white dorsal plumage and the other 3 from white-black dorsal plumage) as sample W-1 library, and 6 black feather bulbs (3 from black dorsal plumage and the other 3 from white-black dorsal plumage) as sample B-1 library. RNA-Seq yielded 5,000,000 raw reads from each library. Low-quality reads (i.e., tags containing only adaptors and ambiguously called bases (reads that has many Ns)) were removed, resulting in 4,887,399 and 4,867,376 clean tags, 217,133 and 235,874 of which were distinct (i.e., non-identical), from white and black feather bulbs, respectively. These distinct clean tags were mapped to 9,009 and 8,498 genes in Ensemble *Gallus gallus* databases, and 1,458 and 1,584 genes in an *Anas platyrhynchos* EST library for W-1 and B-1 libraries, respectively. In total, 10,467 and 10,082 distinct clean tags were mapped to genes, accounting for 4.43% and 4.64% of the total distinct clean tags in the white and black feather bulb RNA libraries, respectively. A summary of sequencing tags and matched genes is shown in Table 1.

**Table 1:** RNA-Seq data summary and annotation results.

RNA-Seq sample		Black feather bulb (B-1)			White feather bulb (W-1)	
Total tags (raw data)		5,000,000			5,000,000	
Clean tags		4,887,399			4,867,376	
Total distinct clean tags		217,133			235,874	
Mapping to gene	Reference		Chicken	Duck	Chicken	Duck
	DCT		8,498	1,584	9,009	1,458
	total		10,082		10,467	
	% of TDCT		4.64%		4.43%	
Total unknown DCT			207,051		225,407	
% unknown of TDCT			95.36%		235,874	

Note: DCT- distinct clean tag; TDCT- Total distinct clean tag; Chicken- *Gallus gallus*; Duck*- Anas platyrhynchos*.

Tag reads analysis showed that more than 83% of the tags were present in 1 to 5 reads, while less than 2% of the tags were present in more than 100 reads. Tags with different numbers of reads between black and white feather bulb libraries matched 5,240 annotated genes. Details of these genes and their related sequence counts in the W-1 and B-1 libraries are listed in Table S1.

### Differentially Expressed Genes in White and Black Feather Bulbs

In this study, a rigorous algorithm was used to identify differentially expressed genes in the two samples based on “The significance of digital gene expression profiles” [26]. A total of 2,642 tags were found to be differentially expressed (DETs). Among these tags, only 186 mapped to annotated genes, yielding 133 differentially expressed genes (log_2_Ratio≥1, P<0.01, FDR<0.001) (see Tables S2 and S3) [26], [27]. Compared to black feather bulbs, white feather bulbs showed 82 downregulated and 51 upregulated genes according to statistical criteria for raw reads and TMP (number of transcripts per million clean tags).

### Gene Ontology Analysis of Differentially Expressed Genes

These 133 genes that are differentially expressed between white and black feather bulbs could be grouped into 94 pathways by gene ontology (GO) analysis. The pathways and differentially expressed genes between the two types of feather bulbs are shown in Table S4. Among the pathways, melanogenesis (c-*Kit*/*Tyr/Tyrp1*) and tyrosine metabolism (*Tyr/Tyrp1*) were directly related to bird plumage pigmentation. A summary of this pathway analysis is shown in Table 2. GO analysis also identified the MAPK (Mitogen-Activated Protein Kinase) signaling pathway, which can link the functions of *Kit* and *Mitf*. Differential expression of the *Mitf* isoforms is dependent on the activation of MEK1-ERK2 in the MAPK signaling pathway [28]. Interestingly, GO analysis also showed enrichment of pathways involving *p53* signaling, apoptosis, Toll-like receptor signaling and immune function. Compared to unpigmented feather bulbs, pigmented feather bulbs have normal melanocytes, in which a series gene cooperated with each other to perform melanogenesis, melanin formation and transportation. Defects occurring in any part of this process may cause feather unpigmentation. The melanogenic pathway which involved in melanogenesis and Tyrosine metabolism was also enriched in the 94 pathways in GO analysis. In addition, we found that pigmented and unpigmented feather bulbs may have differences in many physiological and biochemical processes, including apoptosis, cell cycle, immune response, metabolism, and signaling transduction, etc. It is a complicated regulation network. It could be that genes involved in several processes including melanogenesis and immunity are co-expressed. However, earlier studies showed that some of these pathways may be related to pigmentation [29], which is similar to our results. For example, up-regulation of genes in the Toll like receptor signaling pathway can be associated with melanocytes cell growth and melanogenesis [30]. Our result may provide further evidence for a relationship between TLRs and melanogenesis.

**Table 2:** Digital differential expression analysis of c-*Kit*, *Tyrp1, Tyr* and *Tyrp2* genes between black and white feather bulbs by RNA-Seq.

Gene	Raw-B-1	Raw-W-1	TPM-B-1	TPM-W-1	Log_2_ratio(W-1/B-1)	P-Value			FDR	
c-*Kit*	160	16	32.74	3.29	−3.31489	3.44E-31				5.00E-29
*Tyrp1*	3250	0	664.89	0.01	−16.021			0		0
*Tyr*	184	0	37.65	0.01	−11.8784		5.96E-56			3.48E-54
*Tyrp2*	0	0								

Note: Raw-B-1, Raw data of black feather bulb expression; Raw-W-1, Raw data of white feather bulb expression; TPM-B-1, Normalized expression level of genes in black plumage feather bulb library; TPM-W-1, Normalized expression level of genes in white plumage feather bulb library; Log_2_ ratio(W-1/B-1), log_2_(multiples of differentially expressed genes); P-value corresponds to differential gene expression test; FDR (False Discovery Rate) is used to determine the threshold P-value in multiple tests and analyses by manipulating the FDR value [27].

**Figure 1:**
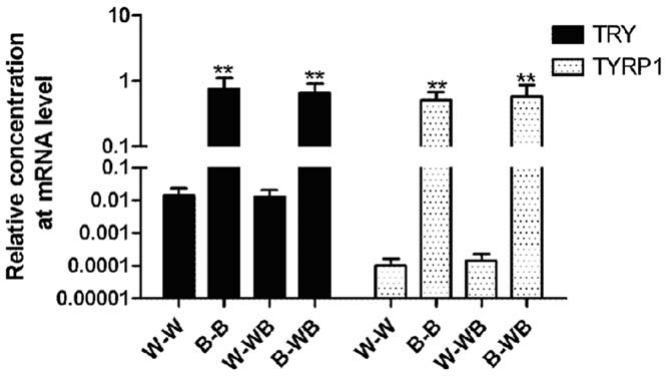
The expression of *Tyrp1* and *Tyr* genes in black and white feather bulb samples from different plumage types.

**Figure 2:**
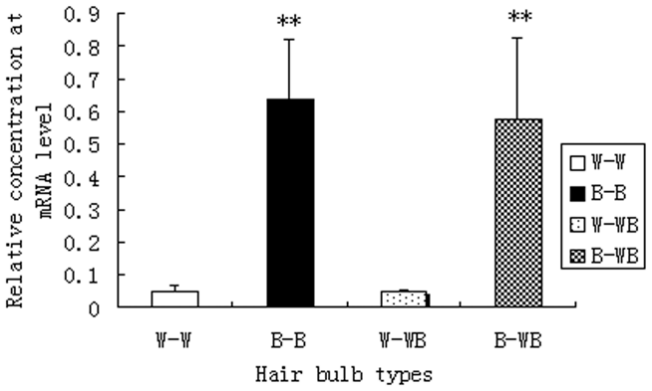
The expression of *c-Kit* gene in black and white feather bulb samples from different plumage types.

### qPCR Confirmation of Differential Gene Expression in White and Black Feather Bulbs from Different Types of Ducks

To confirm the differential gene expression from the RNA-Seq data, we used qPCR to measure the expression of *Tyr, Tyrp1*, and *c-Kit*, three genes in the melanogenesis pathway, in four combinations of feather bulbs and plumage types: W-W (white feather bulb from white plumage), W-WB (white feather bulb from white-black plumage), B-B (black feather bulb from black plumage), and B-WB (black feather bulb from white-black plumage). The results showed that the two critical genes in the melanogenesis pathway, *Tyr* and *Tyrp1*, had almost no expression in white feather bulbs from either white dorsal plumage or white-black dorsal plumage (Figure 1). We also found that *Tyr* and *Tyrp1* were normally expressed in black feather bulbs from either black dorsal plumage or white-black dorsal plumage. The expression or absence of expression of *Tyr* and *Tyrp1* genes indicated the pigmentation status of the feathers, regardless of whether the feather is from ducks with white, black or white-black plumages. *C-Kit* expression was significantly different (P<0.01, Figure 2) between W-W and B-B, or B-WB, as well as between W-WB and B-B, or B-WB samples. In contrast, *c-Kit* expression showed no significant difference between W-W and W-WB or between B-B and B-WB samples. *C-Kit* mRNA expression is approximately 10-fold higher in B-B and B-WB compared to W-W or W-WB samples.

**Figure 3:**
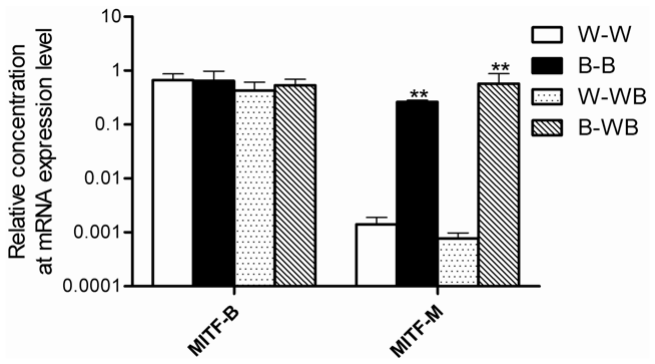
The expression of *Mitf* gene in black and white feather bulb samples from different plumage types.

**Figure 4:**
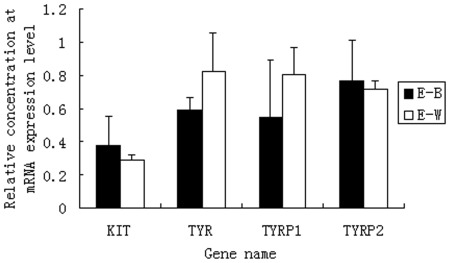
The expression of *Tyrp1*, *Tyr*, *c-Kit* and *Mitf* genes in retina samples from black plumage and white plumage ducks.

**Figure 5:**
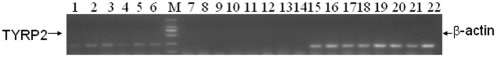
Semi-RT-PCR measurement of *Tyrp2* gene expression in retinas and feather bulbs from ducks with different plumage types. Lanes 1–6: cDNA from 2 replicates of white, white-black and black plumage duck eyes; lanes 7–14: cDNA from 2 replicates of W-W, B-WB, W-WB and B-B feather bulbs; lanes 15–22: *β-actin*, samples are the same as lane 7–14, respectively. M: Marker I, six bands at 600, 500, 400, 300, 200, and 100 bp.

### Expression Comparison of *Mitf*, *Tyr*, *Tyrp1*, *c-Kit* and *Tyrp2* in Retinas and Feather Bulbs

The expression of *Tyr* and *Tyrp1* is regulated by *Mitf*. However, we did not find differential expression of *Mitf* in the RNA-Seq data. To investigate whether *Mitf* showed the same expression pattern as *Tyr* and *Tyrp1*, we performed cloning and qPCR analysis of this gene. The results showed that two isoforms of *Mitf*, M and B, exist. The B isoform was expressed in both black and white feather bulbs, while the M isoform was only expressed in black feather bulbs, regardless of whether they were collected from ducks with pure black or black-white plumage (Figure 3). We also used qPCR to test the relative expression of *Tyr, Tyrp1*, *c-Kit* and *Tyrp2* in retinas, another organ in which melanogenesis occurs. The results showed that all 4 genes were expressed in retinas (Figure 4). Moreover, *Tyrp2* is expressed only in retinas; no expression was detected for this gene in feather bulbs (Figure 5).

## Discussion

Until now, there has been no study using genome-wide expression analysis in duck feather bulbs by RNA- sequencing. Our study offered new information related to gene expression profiles in black and white feather bulbs in the duck. The entire duck genomic sequence is not available; thus, our data analysis was based on the Ensemble *Gallus gallus* database and the *Anas platyrhynchos* EST database. Although duck and chicken coding sequences have high homology (up to 90% for many genes), using the *Gallus gallus* database to match duck sequences can be difficult, as most of the tags are from the 3′UTR of genes. Compared to more than 10 million tags, only 3,000 reference ESTs exist in the *Anas platyrhynchos* EST library, which is too few for annotation. In our study, only 133 genes were identified from 2,642 differentially expressed tags, while most tags did not match any annotated genes. The reasons may include: (1) The short tags were from 3′UTR of the mRNAs; (2) When using the chicken sequences as reference to annotate the genes, the tag will not be annotated to genes if there are 2-bp mismatches as the length of each tag is 17-bp. Thus, only a low proportion of differentially expressed tags could be matched to annotated genes. Also, this method is not sensitive enough to detect genes that are very weakly expressed. Fortunately, three genes in the melanogenesis pathway were identified, indicating that this pathway is crucial for duck plumage color determination.

Skin, coat, and feather color in mammals and birds are determined mainly by 2 melanins, eumelanin and pheomelanin [31]. In human, hair color (or lack thereof, i.e., white hair) is determined by whether the hair bulb has normal biosynthesis of melanin and its subsequent transfer from melanocyte to keratinocytes [32]. In colored feather bulbs, melanin can be synthesized at the first step as described by Korner [33]. For melanogenesis, Tyr, Tyrp1, and Tyrp2 were directly involved in the synthesis of melanins. In this study, Solexa sequencing showed low expression of *Tyr and Tyrp1* in white feather bulbs from ducks with pure white or white-black plumage, while these genes showed normal expression in black feather bulbs from ducks with pure black or white-black plumage. These results demonstrated that a lack of *Tyr and Tyrp1* expression led to a deficiency in the biosynthesis of melanin in white feather bulbs and is the direct cause of white duck plumage. Surprisingly, *Tyrp2* was not expressed in white or black feather bulbs but was expressed in retinas, indicating that this gene may be responsible for retinal pigmentation. This result is similar to that in human [34]. We cloned this gene from duck eye retina mRNA, but the mechanism of its restricted gene expression is not clear.

Previous studies on coat or feather color mainly focused on the effects of nucleotide deletion, mutation, or insertion in single genes. Schmidt [35] found that a single point mutation in exon 1 of the mouse tyrosinase gene caused the dark-eyed albino phenotype. Tobita-Teramoto et al., [3] reported that a six-nucleotide deletion in the tyrosinase coding sequence caused chickens to be albino. Additionally, in chickens, a retroviral insertion in intron 4 of the tyrosinase gene, leading to a lack of exon 5, which encodes the carboxy-terminal membrane spanning domain, caused the recessive white phenotype [4], [36], [37]. In this study, our results showed that the duck *Tyr* gene was expressed normally in retinas from ducks with either black or white plumage. All the ducks in this study had normal, dark retinas. Thus, the *Tyr* gene has normal function ducks with black or white plumage, but the expression in white feather bulbs was suppressed. The genes that inhibit *Tyr* expression could be responsible for plumage color in this population.

It was reported that *Mitf* is a member of the bHLH-leucine zipper transcription factor family and played an important role in the development of retinal cells, mast cells, osteoclasts and melanocytes [38]. Alleles of *Mitf* have been associated with coat color in dogs [7] and mice [39], [40], as well as with plumage color in quail [41]. Minvielle [10] demonstrated that a 2-bp deletion in exon 11 of *Mitf* caused white plumage in Japanese quail. In this study, in contrast to Japanese quail, the *Mitf*-M isoform did not show expression in white plumage feather bulbs, although both *Mitf*-M and *Mitf*-B isoforms were normally expressed in retinas and black plumage feather bulbs. The difference in *Mitf*-M expression between white and black feather bulbs in this study suggests that *Mitf*-M is involved in determining feather pigmentation in the duck through either cis or trans acting regulatory elements as opposed to non-synonymous coding variants like in the quail. The expression pattern of the duck *Pmel17* is the same as *Mitf-*M, *Tyr, Tyrp1*. *Pmel17* plays a central role in the biogenesis of melanosomes. Studies in other animals showed that this gene is involved in the maturation of melanosomes [42]. Plumage variants in chicken are associated with insertion/deletion polymorphisms in the *Pmel17* gene [23], [43], [44]. Also, silver coat color in horse is associated with a missense mutation in the *Pmel17* gene [45]. Our study demonstrated *Pmel17* may also play roles in duck pigmentation.

Although little is known about the role of *c-Kit* expression in the regulation of plumage color in birds, the roles of *Scf* and *c-Kit* signaling in melanoblast or melanocyte migration, proliferation and differentiation during embryogenesis and maintaining postnatal cutaneous melanogenesis were reported in mammals [13], [46], [47]. Additionally, the function of *c-Kit* in feather follicle melanogenesis [46], [48], [49] and the maintenance of human hair pigmentation [50] have been widely studied in various models. Nucleotide deletions from introns [14] and copy-number variation [51]–[53] of *c-Kit* were associated with pig coat color. *C-Kit* mutations in horses were associated with white coat color [16], [54]–[56], and exon skipping in the *c-Kit* gene in horses causes a Sabino spotting pattern [57]. Furthermore, *c-Kit* is a candidate for white spotting in cats [58], and the *c-Kit* signaling pathway is involved in post-developmental processes of mature cells [59]. Taken together, the *c-Kit* gene plays a critical role in animal coat color and is specifically associated with ‘white’. In this study, we found there was no significant difference in *c-Kit* expression in retinas from ducks with white or black plumage. W-W, B-B, W-WB, and B-WB feather bulbs all showed expression of the *c-Kit* gene, although the expression level in black feather bulbs was 10-fold higher than that in white feather bulbs. In white feather bulbs, this basal level of *c-Kit* expression may be able to maintain cell proliferation and differentiation but is not sufficient to promote pigmentation, although there is no *in vitro* confirmation work. In contrast, *c-Kit* expression in black feather bulbs is 10 times higher than that in white feather bulbs, allowing for cell proliferation and differentiation as well as maintenance of postnatal melanogenesis. It is possible that the lower level of *c-Kit* expression in the white feather bulb is a downstream consequence of few or no active melanocytes in the feather bulb, but not a genetic lesion at the *c-Kit* locus.

### Conclusion

Plumage color variation in *Kaiya-Liancheng* F_2_ ducks was determined by whether melanin can be synthesized in the feather bulb. Our results provide solid evidence on some of the functional players in feather pigmentation in the duck, e.g. upregulation of *c-Kit* and *Mitf* in black feather bulbs may be responsible for black plumage formation.

**Figure 6:**
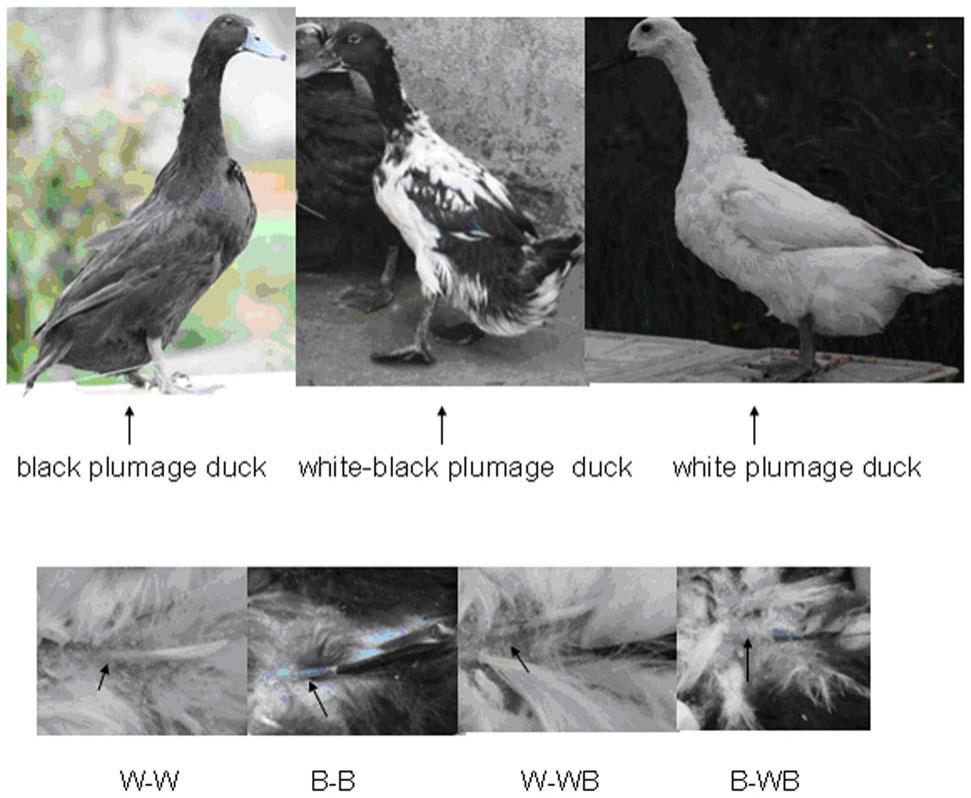
Plumage types and feather bulbs used in this study. A: Three duck plumage types; B: Feather bulbs from four plumage types, W-W (white feather bulb from white plumage), B-B (black feather bulb from black plumage), W-WB (white feather bulb from white-black plumage), B-WB (black feather bulb from white-black plumage).

## Materials and Methods

### Experimental Animals

The genetic background of experimental ducks was described by Gong et al., [25]. Three white plumage ducks, three black plumage ducks and three white-black plumage ducks were randomly selected from a population of the *Kaiya-Liancheng* F_2_ generation. The three plumage patterns are shown in Figure 6. Three feather bulbs from the same individual were pooled as one sample. The white feather bulbs from white duck back were marked as W-W, whereas the black feather bulbs from black duck back were marked as B-B. White feather bulbs from white-black duck back were marked as W-WB and black feather bulbs from white-black duck back were marked as B-WB, respectively. The four feather bulb types are shown in Figure 6. All research involving animals were conducted according to the regulation (No. 5 proclaim of the Standing Committee of Hubei People’s Congress) approved by the Standing Committee of Hubei People’s Congress, and the ethics committee of Huazhong Agricultural University, P. R. China. The approved permit number for this study is “HBAC20091138”.

**Table 3:** Primers used in Semi-RT-PCR and qPCR.

Gene	Primers	Sequence (5′- 3′)	Size (bp)	AT	Function
*β-actin*	β-actin-Fβ-actin-R	AACTGGGATGACATGGAGAAGA ATGGCTGGGGTGTTGAAGGT	189	60°C	Semi-RT PCR & qPCR
c-*Kit*	c-*Kit*-Fc-*Kit*-R	GCTGATGCTGCCAATGAGT TTTGCCACCTGGTAAGAGA	151	60°C	qPCR
*Tyr*	E_3_-F_1425_E_4_NR	TTACATGGTCCCCTTTATTC CAATCACAGCTGCACCAACC	182	60°C	qPCR
*Tyrp1*	F_1250_R_1439_	AATGAGATGTTTGTTACTG ACTGATCAGTGAGAAGAGG	208	60°C	qPCR
*Tyrp2*	F_1496_R_1703_	CACCTATGCCATTGACCTGCC AGCAAGGAAACGAAGCAAGGG	228	60°C	qPCR
*Mitf*-B	BF_383_MBR_220_	CCCAGTTCATGCAGCAGAGAGT CCAGGCGGCATGACATGATCAC	268	60°C	Semi-RT-PCR
*Mitf*-M	MF_16_MBR_220_	TGCAGTCACTTCTCTCACAACC CCAGGCGGCATGACATGATCAC	226	60°C	qPCR

Note: AT = Annealing temperature.

### Total RNA Extraction from Feather Bulbs and RT-PCR

Feather bulbs were put into 2-mL tubes containing 1 mL TRIzol reagent (Invitrogen, San Diego, CA). One ceramic bead was added immediately to each tube. The tubes were then ground for 30 seconds by EASY GRIND. Total RNA was extracted according to the manufacturer’s protocol. The quality and quantity of RNA samples were checked by Spectrophotometer ND-1000 (Nano-Drop) and denaturing agarose gel electrophoresis. All RNA samples were treated with DNAse-I for later use.

### RNA-Seq, Data Mining and Gene Ontology Analysis

Solexa sequencing of W-W and B-B pooled RNA was conducted in BGI, Shenzhen. Three databases were employed for sequence analysis, the Ensemble *Gallus gallus* databases, reference gene (ftp.ensembl.org/pub/release-59/fasta/gallus_gallus/cdna/Gallus gallus. WASHUC2.59.cdna. all. fa.gz), reference genomic DNA (ftp://ftp.ensembl.org/pub/release-59/fasta/gallus_gallus/dna), and the *Anas platyrhynchos* EST database (http://www.ncbi.nlm.nih.gov/nucest/?term=Anas%20platyrhynchos). In this study, a rigorous algorithm has been developed to identify differentially expressed genes between two samples by BGI based on “The significance of digital gene expression profiles” [26]. We used a P-value corresponding to a differential gene expression test at statistically significant levels [27]. “FDR (False Discovery Rate) ≤0.001 and the absolute value of log2Ratio≥1” were used to identify DEGs (Different Expression Genes) and DETs (Different Expression Tags). Pathways of differentially expressed genes were analyzed by DAVID v6.7. Gene function classification was conducted using the Gene Ontology FAT set term.

### *c-Kit, Tyr, Tyrp1* and *Tyrp2* Gene Expression in Feather Bulb and Retina Samples by qPCR

To confirm the differential expression of genes revealed by RNA-Seq, the expression of genes in the melanogenesis pathway, including *c-Kit, Tyr* and *Tyrp1*, was measured by qPCR. In addition, the expression of *Mitf* and *Tyrp2* was measured because they are directly involved in melanin biosynthesis. *β*-actin was used as a reference control. qPCR analysis was performed on Roche lightercycler® 480, using lightercycler® 480 SYBR Green I master detection reagents (Roche Diagnostics 11367523). All reactions were performed in triplicate within each PCR assay and under the same cycling conditions: denaturation at 95°C for 3 min, followed by 40 cycles of amplification (95°C for 20 s, 60°C for 20 s, and 72°C for 20 s) with a single acquisition of fluorescence at the end of the extension step. Melt curve analysis was performed over a range of 55∼95°C to verify single product generation at the end of the assay. qPCR data analysis was performed with the Light Cycler analysis software. Relative quantification analyses were performed in EXCEL using the comparative CT method. Comparisons between qPCR data sets were made with Student’s t-test. Differences were considered significant if P<0.05. Further, semi-RT-PCR measurements of *Tyrp2* gene expression in retinas and feather bulbs from ducks with different plumage types were also conducted. All primers information is shown in Table 3.

## Supporting Information

Table S1 **The information of genes that expressed in duck feather bulbs that have different reads.**
(XLS)

Table S2 **Differentially expressed tags between white and black plumage feather bulb libraries.**
(XLS)

Table S3 **Differentially expressed genes between white and black plumage feather bulb libraries.**
(XLS)

Table S4 **Gene Ontology analysis of the differentially expressed genes.**
(DOC)
