# Supplementary material for: Identification of Genes Related to White and Black Plumage Formation by RNA-Seq from White and Black Feather Bulbs in Ducks
Source: PLoS One. 2012 May 15;7(5):e36592. doi: 10.1371/journal.pone.0036592 (PMC3352928; doi:10.1371/journal.pone.0036592)
Supplement: Table S4 — Gene Ontology analysis of the differentially expressed genes. (DOC) [file pone.0036592.s004.doc]

Supplementary Table S4: The result of solexa sequencing about Gene Ontology for pathway and differentially expressed genes

|  | Pathway | **Differentially expressed genes** |
| --- | --- | --- |
| 1 | [Ribosome](../../../../E:%5C实验结果分类%5C华大结果1-2%5C鸡鸭合并%5Cpathway%5CB-1vsW-1_map%5Cmap03010.html) | gi|109971394|gb|DR765069.1|DR765069, gi|109973653|gb|DR766277.1|DR766277, gi|109971892|gb|DR765345.1|DR765345, gi|109969514|gb|DR764053.1|DR764053, gi|109971421|gb|DR765085.1|DR765085, gi|109972942|gb|DR765926.1|DR765926, ENSGALT00000019972 |
| 2 | [NOD-like receptor signaling pathway](../../../../E:%5C实验结果分类%5C华大结果1-2%5C鸡鸭合并%5Cpathway%5CB-1vsW-1_map%5Cmap04621.html) | ENSGALT00000005241, ENSGALT00000013396, ENSGALT00000011181 |
| 3 | [Basal transcription factors](../../../../E:%5C实验结果分类%5C华大结果1-2%5C鸡鸭合并%5Cpathway%5CB-1vsW-1_map%5Cmap03022.html) | ENSGALT00000008282,ENSGALT00000006017 |
| 4 | [Endocytosis](../../../../E:%5C实验结果分类%5C华大结果1-2%5C鸡鸭合并%5Cpathway%5CB-1vsW-1_map%5Cmap04144.html) | gi|109973330|gb|DR766140.1|DR766140, ENSGALT00000004943, ENSGALT00000022571, ENSGALT00000018060, ENSGALT00000008250, ENSGALT00000008282 |
| 5 | Aldosterone-regulated sodium reabsorption | ENSGALT00000031766, ENSGALT00000004364 |
| 6 | [Tyrosine metabolism](../../../../E:%5C实验结果分类%5C华大结果1-2%5C鸡鸭合并%5Cpathway%5CB-1vsW-1_map%5Cmap00350.html) | DUCK-TYRP1, DUCK-TYR |
| 7 | [Cardiac muscle contraction](../../../../E:%5C实验结果分类%5C华大结果1-2%5C鸡鸭合并%5Cpathway%5CB-1vsW-1_map%5Cmap04260.html) | gi|109970307|gb|DR764479.1|DR764479, ENSGALT00000007087, ENSGALT00000009092, ENSGALT00000004364 |
| 8 | [Huntington's disease](../../../../E:%5C实验结果分类%5C华大结果1-2%5C鸡鸭合并%5Cpathway%5CB-1vsW-1_map%5Cmap05016.html) | gi|109970307|gb|DR764479.1|DR764479, ENSGALT00000013396, ENSGALT00000008250, ENSGALT00000007087 |
| 9 | [Melanogenesis](../../../../E:%5C实验结果分类%5C华大结果1-2%5C鸡鸭合并%5Cpathway%5CB-1vsW-1_map%5Cmap04916.html) | DUCK-TYRP1, DUCK-TYR, ENSGALT00000022571 |
| 10 | [mTOR signaling pathway](../../../../E:%5C实验结果分类%5C华大结果1-2%5C鸡鸭合并%5Cpathway%5CB-1vsW-1_map%5Cmap04150.html) | ENSGALT00000008872, ENSGALT00000019364 |
| 11 | [Lysosome](../../../../E:%5C实验结果分类%5C华大结果1-2%5C鸡鸭合并%5Cpathway%5CB-1vsW-1_map%5Cmap04142.html) | gi|109970993|gb|DR764852.1|DR764852, ENSGALT00000018986, ENSGALT00000008250 |
| 12 | [Acute myeloid leukemia](../../../../E:%5C实验结果分类%5C华大结果1-2%5C鸡鸭合并%5Cpathway%5CB-1vsW-1_map%5Cmap05221.html) | ENSGALT00000005241, ENSGALT00000022571 |
| 13 | [Glyoxylate and dicarboxylate metabolism](../../../../E:%5C实验结果分类%5C华大结果1-2%5C鸡鸭合并%5Cpathway%5CB-1vsW-1_map%5Cmap00630.html) | ENSGALT00000003016 |
| 14 | [Nitrogen metabolism](../../../../E:%5C实验结果分类%5C华大结果1-2%5C鸡鸭合并%5Cpathway%5CB-1vsW-1_map%5Cmap00910.html) | ENSGALT00000013873 |
| 15 | [Protein export](../../../../E:%5C实验结果分类%5C华大结果1-2%5C鸡鸭合并%5Cpathway%5CB-1vsW-1_map%5Cmap03060.html) | gi|109973169|gb|DR766053.1|DR766053 |
| 16 | Glycosaminoglycan biosynthesis - heparan sulfate | ENSGALT00000010110  (no map in kegg database) |
| 17 | [Proximal tubule bicarbonate reclamation](../../../../E:%5C实验结果分类%5C华大结果1-2%5C鸡鸭合并%5Cpathway%5CB-1vsW-1_map%5Cmap04964.html) | ENSGALT00000004364 |
| 18 | [Salivary secretion](../../../../E:%5C实验结果分类%5C华大结果1-2%5C鸡鸭合并%5Cpathway%5CB-1vsW-1_map%5Cmap04970.html) | ENSGALT00000006816, ENSGALT00000004364 |
| 19 | [Adipocytokine signaling pathway](../../../../E:%5C实验结果分类%5C华大结果1-2%5C鸡鸭合并%5Cpathway%5CB-1vsW-1_map%5Cmap04920.html) | ENSGALT00000017891, ENSGALT00000005241 |
| 20 | [Pentose phosphate pathway](../../../../E:%5C实验结果分类%5C华大结果1-2%5C鸡鸭合并%5Cpathway%5CB-1vsW-1_map%5Cmap00030.html) | gi|109973066|gb|DR765996.1|DR765996 |
| 21 | [Spliceosome](../../../../E:%5C实验结果分类%5C华大结果1-2%5C鸡鸭合并%5Cpathway%5CB-1vsW-1_map%5Cmap03040.html) | ENSGALT00000011020, ENSGALT00000031818, ENSGALT00000005586 |
| 22 | [Citrate cycle (TCA cycle)](../../../../E:%5C实验结果分类%5C华大结果1-2%5C鸡鸭合并%5Cpathway%5CB-1vsW-1_map%5Cmap00020.html) | ENSGALT00000003016 |
| 23 | [DNA replication](../../../../E:%5C实验结果分类%5C华大结果1-2%5C鸡鸭合并%5Cpathway%5CB-1vsW-1_map%5Cmap03030.html) | ENSGALT00000027483 |
| 24 | [Alanine, aspartate and glutamate metabolism](../../../../E:%5C实验结果分类%5C华大结果1-2%5C鸡鸭合并%5Cpathway%5CB-1vsW-1_map%5Cmap00250.html) | ENSGALT00000013873 |
| 25 | [RNA polymerase](../../../../E:%5C实验结果分类%5C华大结果1-2%5C鸡鸭合并%5Cpathway%5CB-1vsW-1_map%5Cmap03020.html) | ENSGALT00000008663 |
| 26 | [Oxidative phosphorylation](../../../../E:%5C实验结果分类%5C华大结果1-2%5C鸡鸭合并%5Cpathway%5CB-1vsW-1_map%5Cmap00190.html) | gi|109970307|gb|DR764479.1|DR764479, ENSGALT00000007087 |
| 27 | [Antigen processing and presentation](../../../../E:%5C实验结果分类%5C华大结果1-2%5C鸡鸭合并%5Cpathway%5CB-1vsW-1_map%5Cmap04612.html) | gi|109970993|gb|DR764852.1|DR764852, ENSGALT00000005355 |
| 28 | [Small cell lung cancer](../../../../E:%5C实验结果分类%5C华大结果1-2%5C鸡鸭合并%5Cpathway%5CB-1vsW-1_map%5Cmap05222.html) | ENSGALT00000022704, ENSGALT00000005241 |
| 29 | [Cytosolic DNA-sensing pathway](../../../../E:%5C实验结果分类%5C华大结果1-2%5C鸡鸭合并%5Cpathway%5CB-1vsW-1_map%5Cmap04623.html) | ENSGALT00000005241 |
| 30 | [Tryptophan metabolism](../../../../E:%5C实验结果分类%5C华大结果1-2%5C鸡鸭合并%5Cpathway%5CB-1vsW-1_map%5Cmap00380.html) | ENSGALT00000020299 |
| 31 | [Arrhythmogenic right ventricular cardiomyopathy (ARVC)](../../../../E:%5C实验结果分类%5C华大结果1-2%5C鸡鸭合并%5Cpathway%5CB-1vsW-1_map%5Cmap05412.html) | ENSGALT00000022704, ENSGALT00000020869 |
| 32 | [Fructose and mannose metabolism](../../../../E:%5C实验结果分类%5C华大结果1-2%5C鸡鸭合并%5Cpathway%5CB-1vsW-1_map%5Cmap00051.html) | gi|109973066|gb|DR765996.1|DR765996 |
| 33 | [Parkinson's disease](../../../../E:%5C实验结果分类%5C华大结果1-2%5C鸡鸭合并%5Cpathway%5CB-1vsW-1_map%5Cmap05012.html) | gi|109970307|gb|DR764479.1|DR764479, ENSGALT00000007087 |
| 34 | [Oocyte meiosis](../../../../E:%5C实验结果分类%5C华大结果1-2%5C鸡鸭合并%5Cpathway%5CB-1vsW-1_map%5Cmap04114.html) | ENSGALT00000009864, ENSGALT00000006588 |
| 35 | [Axon guidance](../../../../E:%5C实验结果分类%5C华大结果1-2%5C鸡鸭合并%5Cpathway%5CB-1vsW-1_map%5Cmap04360.html) | ENSGALT00000037665, ENSGALT00000037253, ENSGALT00000008440 |
| 36 | [Cell cycle](../../../../E:%5C实验结果分类%5C华大结果1-2%5C鸡鸭合并%5Cpathway%5CB-1vsW-1_map%5Cmap04110.html) | ENSGALT00000009864, ENSGALT00000006588 |
| 37 | [Chagas disease](../../../../E:%5C实验结果分类%5C华大结果1-2%5C鸡鸭合并%5Cpathway%5CB-1vsW-1_map%5Cmap05142.html) | ENSGALT00000000314, ENSGALT00000005241 |
| 38 | [Ether lipid metabolism](../../../../E:%5C实验结果分类%5C华大结果1-2%5C鸡鸭合并%5Cpathway%5CB-1vsW-1_map%5Cmap00565.html) | ENSGALT00000005659 |
| 39 | [Dorso-ventral axis formation](../../../../E:%5C实验结果分类%5C华大结果1-2%5C鸡鸭合并%5Cpathway%5CB-1vsW-1_map%5Cmap04320.html) | ENSGALT00000000717 |
| 40 | Metabolic pathways (no map in kegg database) | DUCK-TYRP1, DUCK-TYR, gi|109970307|gb|DR764479.1|DR764479, gi|109973066|gb|DR765996.1|DR765996, ENSGALT00000008663, ENSGALT00000005659, ENSGALT00000013873, ENSGALT00000007087, ENSGALT00000020299, ENSGALT00000003016, ENSGALT00000004318 |
| 41 | [Arginine and proline metabolism](../../../../E:%5C实验结果分类%5C华大结果1-2%5C鸡鸭合并%5Cpathway%5CB-1vsW-1_map%5Cmap00330.html) | ENSGALT00000013873 |
| 42 | [Protein processing in endoplasmic reticulum](../../../../E:%5C实验结果分类%5C华大结果1-2%5C鸡鸭合并%5Cpathway%5CB-1vsW-1_map%5Cmap04141.html) | ENSGALT00000019309, ENSGALT00000016555 |
| 43 | [Pyruvate metabolism](../../../../E:%5C实验结果分类%5C华大结果1-2%5C鸡鸭合并%5Cpathway%5CB-1vsW-1_map%5Cmap00620.html) | ENSGALT00000003016 |
| 44 | [Type I diabetes mellitus](../../../../E:%5C实验结果分类%5C华大结果1-2%5C鸡鸭合并%5Cpathway%5CB-1vsW-1_map%5Cmap04940.html) | ENSGALT00000013137 |
| 45 | [RIG-I-like receptor signaling pathway](../../../../E:%5C实验结果分类%5C华大结果1-2%5C鸡鸭合并%5Cpathway%5CB-1vsW-1_map%5Cmap04622.html) | ENSGALT00000005241 |
| 46 | [Ubiquitin mediated proteolysis](../../../../E:%5C实验结果分类%5C华大结果1-2%5C鸡鸭合并%5Cpathway%5CB-1vsW-1_map%5Cmap04120.html) | ENSGALT00000038423, ENSGALT00000008016 |
| 47 | [Vascular smooth muscle contraction](../../../../E:%5C实验结果分类%5C华大结果1-2%5C鸡鸭合并%5Cpathway%5CB-1vsW-1_map%5Cmap04270.html) | ENSGALT00000018836, ENSGALT00000006816, ENSGALT00000009092 |
| 48 | [Inositol phosphate metabolism](../../../../E:%5C实验结果分类%5C华大结果1-2%5C鸡鸭合并%5Cpathway%5CB-1vsW-1_map%5Cmap00562.html) | ENSGALT00000004318 |
| 49 | [Glycolysis / Gluconeogenesis](../../../../E:%5C实验结果分类%5C华大结果1-2%5C鸡鸭合并%5Cpathway%5CB-1vsW-1_map%5Cmap00010.html) | gi|109973066|gb|DR765996.1|DR765996 |
| 50 | [Glycerophospholipid metabolism](../../../../E:%5C实验结果分类%5C华大结果1-2%5C鸡鸭合并%5Cpathway%5CB-1vsW-1_map%5Cmap00564.html) | ENSGALT00000005659 |
| 51 | [Alzheimer's disease](../../../../E:%5C实验结果分类%5C华大结果1-2%5C鸡鸭合并%5Cpathway%5CB-1vsW-1_map%5Cmap05010.html) | gi|109970307|gb|DR764479.1|DR764479, ENSGALT00000007087 |
| 52 | [RNA degradation](../../../../E:%5C实验结果分类%5C华大结果1-2%5C鸡鸭合并%5Cpathway%5CB-1vsW-1_map%5Cmap03018.html) | ENSGALT00000013137 |
| 53 | [Riboflavin metabolism](../../../../E:%5C实验结果分类%5C华大结果1-2%5C鸡鸭合并%5Cpathway%5CB-1vsW-1_map%5Cmap00740.html) | DUCK-TYR |
| 54 | [Epithelial cell signaling in Helicobacter pylori infection](../../../../E:%5C实验结果分类%5C华大结果1-2%5C鸡鸭合并%5Cpathway%5CB-1vsW-1_map%5Cmap05120.html) | ENSGALT00000005241 |
| 55 | [ECM-receptor interaction](../../../../E:%5C实验结果分类%5C华大结果1-2%5C鸡鸭合并%5Cpathway%5CB-1vsW-1_map%5Cmap04512.html) | ENSGALT00000022704, ENSGALT00000006816 |
| 56 | [Renal cell carcinoma](../../../../E:%5C实验结果分类%5C华大结果1-2%5C鸡鸭合并%5Cpathway%5CB-1vsW-1_map%5Cmap05211.html) | ENSGALT00000019364 |
| 57 | [Pancreatic cancer](../../../../E:%5C实验结果分类%5C华大结果1-2%5C鸡鸭合并%5Cpathway%5CB-1vsW-1_map%5Cmap05212.html) | ENSGALT00000005241 |
| 58 | [Progesterone-mediated oocyte maturation](../../../../E:%5C实验结果分类%5C华大结果1-2%5C鸡鸭合并%5Cpathway%5CB-1vsW-1_map%5Cmap04914.html) | ENSGALT00000009864 |
| 59 | [Long-term depression](../../../../E:%5C实验结果分类%5C华大结果1-2%5C鸡鸭合并%5Cpathway%5CB-1vsW-1_map%5Cmap04730.html) | ENSGALT00000006816 |
| 60 | [Cytokine-cytokine receptor interaction](../../../../E:%5C实验结果分类%5C华大结果1-2%5C鸡鸭合并%5Cpathway%5CB-1vsW-1_map%5Cmap04060.html) | ENSGALT00000022571, ENSGALT00000010256 |
| 61 | [Pyrimidine metabolism](../../../../E:%5C实验结果分类%5C华大结果1-2%5C鸡鸭合并%5Cpathway%5CB-1vsW-1_map%5Cmap00240.html) | ENSGALT00000008663 |
| 62 | [p53 signaling pathway](../../../../E:%5C实验结果分类%5C华大结果1-2%5C鸡鸭合并%5Cpathway%5CB-1vsW-1_map%5Cmap04115.html) | ENSGALT00000038423 |
| 63 | [Chronic myeloid leukemia](../../../../E:%5C实验结果分类%5C华大结果1-2%5C鸡鸭合并%5Cpathway%5CB-1vsW-1_map%5Cmap05220.html) | ENSGALT00000005241 |
| 64 | [Chemokine signaling pathway](../../../../E:%5C实验结果分类%5C华大结果1-2%5C鸡鸭合并%5Cpathway%5CB-1vsW-1_map%5Cmap04062.html) | ENSGALT00000005241, ENSGALT00000010256 |
| 65 | [Apoptosis](../../../../E:%5C实验结果分类%5C华大结果1-2%5C鸡鸭合并%5Cpathway%5CB-1vsW-1_map%5Cmap04210.html) | ENSGALT00000005241 |
| 66 | [Viral myocarditis](../../../../E:%5C实验结果分类%5C华大结果1-2%5C鸡鸭合并%5Cpathway%5CB-1vsW-1_map%5Cmap05416.html) | ENSGALT00000022704, ENSGALT00000014159, ENSGALT00000009092 |
| 67 | [Toll-like receptor signaling pathway](../../../../E:%5C实验结果分类%5C华大结果1-2%5C鸡鸭合并%5Cpathway%5CB-1vsW-1_map%5Cmap04620.html) | ENSGALT00000005241 |
| 68 | [Long-term potentiation](../../../../E:%5C实验结果分类%5C华大结果1-2%5C鸡鸭合并%5Cpathway%5CB-1vsW-1_map%5Cmap04720.html) | ENSGALT00000018836 |
| 69 | [Gap junction](../../../../E:%5C实验结果分类%5C华大结果1-2%5C鸡鸭合并%5Cpathway%5CB-1vsW-1_map%5Cmap04540.html) | ENSGALT00000006816 |
| 70 | [Pathways in cancer](../../../../E:%5C实验结果分类%5C华大结果1-2%5C鸡鸭合并%5Cpathway%5CB-1vsW-1_map%5Cmap05200.html) | ENSGALT00000022704, ENSGALT00000005241, ENSGALT00000022571, ENSGALT00000019364 |
| 71 | [ErbB signaling pathway](../../../../E:%5C实验结果分类%5C华大结果1-2%5C鸡鸭合并%5Cpathway%5CB-1vsW-1_map%5Cmap04012.html) | ENSGALT00000004943 |
| 72 | [Prostate cancer](../../../../E:%5C实验结果分类%5C华大结果1-2%5C鸡鸭合并%5Cpathway%5CB-1vsW-1_map%5Cmap05215.html) | ENSGALT00000005241 |
| 73 | [Phosphatidylinositol signaling system](../../../../E:%5C实验结果分类%5C华大结果1-2%5C鸡鸭合并%5Cpathway%5CB-1vsW-1_map%5Cmap04070.html) | ENSGALT00000004318 |
| 74 | [Gastric acid secretion](../../../../E:%5C实验结果分类%5C华大结果1-2%5C鸡鸭合并%5Cpathway%5CB-1vsW-1_map%5Cmap04971.html) | ENSGALT00000004364 |
| 75 | [Shigellosis](../../../../E:%5C实验结果分类%5C华大结果1-2%5C鸡鸭合并%5Cpathway%5CB-1vsW-1_map%5Cmap05131.html) | ENSGALT00000005241 |
| 76 | [Hypertrophic cardiomyopathy (HCM)](../../../../E:%5C实验结果分类%5C华大结果1-2%5C鸡鸭合并%5Cpathway%5CB-1vsW-1_map%5Cmap05410.html) | ENSGALT00000022704, ENSGALT00000009092 |
| 77 | [T cell receptor signaling pathway](../../../../E:%5C实验结果分类%5C华大结果1-2%5C鸡鸭合并%5Cpathway%5CB-1vsW-1_map%5Cmap04660.html) | ENSGALT00000005241 |
| 78 | [Focal adhesion](../../../../E:%5C实验结果分类%5C华大结果1-2%5C鸡鸭合并%5Cpathway%5CB-1vsW-1_map%5Cmap04510.html) | ENSGALT00000018836, ENSGALT00000022704, ENSGALT00000006816 |
| 79 | [Bacterial invasion of epithelial cells](../../../../E:%5C实验结果分类%5C华大结果1-2%5C鸡鸭合并%5Cpathway%5CB-1vsW-1_map%5Cmap05100.html) | ENSGALT00000008250 |
| 80 | [Amoebiasis](../../../../E:%5C实验结果分类%5C华大结果1-2%5C鸡鸭合并%5Cpathway%5CB-1vsW-1_map%5Cmap05146.html) | ENSGALT00000022704, ENSGALT00000006816 |
| 81 | [Regulation of actin cytoskeleton](../../../../E:%5C实验结果分类%5C华大结果1-2%5C鸡鸭合并%5Cpathway%5CB-1vsW-1_map%5Cmap04810.html) | ENSGALT00000018836, ENSGALT00000008413, ENSGALT00000009092 |
| 82 | Pathogenic Escherichia coli infection | ENSGALT00000006588 (no map in kegg database) |
| 83 | [MAPK signaling pathway](../../../../E:%5C实验结果分类%5C华大结果1-2%5C鸡鸭合并%5Cpathway%5CB-1vsW-1_map%5Cmap04010.html) | ENSGALT00000037665, ENSGALT00000005241 |
| 84 | [Neurotrophin signaling pathway](../../../../E:%5C实验结果分类%5C华大结果1-2%5C鸡鸭合并%5Cpathway%5CB-1vsW-1_map%5Cmap04722.html) | ENSGALT00000006588 |
| 85 | Insulin signaling pathway | ENSGALT00000004318 (no map in kegg database) |
| 86 | [Purine metabolism](../../../../E:%5C实验结果分类%5C华大结果1-2%5C鸡鸭合并%5Cpathway%5CB-1vsW-1_map%5Cmap00230.html) | ENSGALT00000008663 |
| 87 | [Tight junction](../../../../E:%5C实验结果分类%5C华大结果1-2%5C鸡鸭合并%5Cpathway%5CB-1vsW-1_map%5Cmap04530.html) | ENSGALT00000000314, ENSGALT00000009092 |
| 88 | [Phagosome](../../../../E:%5C实验结果分类%5C华大结果1-2%5C鸡鸭合并%5Cpathway%5CB-1vsW-1_map%5Cmap04145.html) | gi|109970993|gb|DR764852.1|DR764852, ENSGALT00000008282 |
| 89 | [Olfactory transduction](../../../../E:%5C实验结果分类%5C华大结果1-2%5C鸡鸭合并%5Cpathway%5CB-1vsW-1_map%5Cmap04740.html) | ENSGALT00000006816 |
| 90 | [Hematopoietic cell lineage](../../../../E:%5C实验结果分类%5C华大结果1-2%5C鸡鸭合并%5Cpathway%5CB-1vsW-1_map%5Cmap04640.html) | ENSGALT00000022571 |
| 91 | [Dilated cardiomyopathy](../../../../E:%5C实验结果分类%5C华大结果1-2%5C鸡鸭合并%5Cpathway%5CB-1vsW-1_map%5Cmap05414.html) | ENSGALT00000022704, ENSGALT00000009092 |
| 92 | [Systemic lupus erythematosus](../../../../E:%5C实验结果分类%5C华大结果1-2%5C鸡鸭合并%5Cpathway%5CB-1vsW-1_map%5Cmap05322.html) | ENSGALT00000009951 |
| 93 | [B cell receptor signaling pathway](../../../../E:%5C实验结果分类%5C华大结果1-2%5C鸡鸭合并%5Cpathway%5CB-1vsW-1_map%5Cmap04662.html) | ENSGALT00000005241 |
| 94 | [Calcium signaling pathway](../../../../E:%5C实验结果分类%5C华大结果1-2%5C鸡鸭合并%5Cpathway%5CB-1vsW-1_map%5Cmap04020.html) | ENSGALT00000004943 |
